# Tutorial: Speech Assessment for Multilingual Children Who Do Not Speak the Same Language(s) as the Speech-Language Pathologist

**DOI:** 10.1044/2017_AJSLP-15-0161

**Published:** 2017-08-15

**Authors:** Sharynne McLeod, Sarah Verdon, The International Expert Panel on Multilingual Children's Speech, Elise Baker, Martin J. Ball, Elaine Ballard, Avivit Ben David, B. May Bernhardt, Daniel Bérubé, Mirjam Blumenthal, Caroline Bowen, Françoise Brosseau-Lapré, Ferenc Bunta, Kathryn Crowe, Madalena Cruz-Ferreira, Barbara Davis, Annette Fox-Boyer, Christina Gildersleeve-Neumann, Helen Grech, Brian Goldstein, Anne Hesketh, Suzanne Hopf, Minjung Kim, Sari Kunnari, Andrea MacLeod, Jane McCormack, Þóra (Thora) Másdóttir, Glenda Mason, Sarah Masso, Sandra Neumann, Martina Ozbič, Michelle Pascoe, Giang Pham, Rosario Román, Yvan Rose, Susan Rvachew, Tuula Savinainen-Makkonen, Seyhun Topbaş, Nancy Scherer, Jane Speake, Joseph P. Stemberger, Isao Ueda, Karla N. Washington, Carol Westby, A. Lynn Williams, Yvonne Wren, Krisztina Zajdó, Natalia Zharkova

**Affiliations:** a Charles Sturt University, Bathurst, New South Wales, Australia; b International Expert Panel on Multilingual Children's Speech, Bathurst, New South Wales, Australia

## Abstract

**Purpose:**

The aim of this tutorial is to support speech-language pathologists (SLPs) undertaking assessments of multilingual children with suspected speech sound disorders, particularly children who speak languages that are not shared with their SLP.

**Method:**

The tutorial was written by the International Expert Panel on Multilingual Children's Speech, which comprises 46 researchers (SLPs, linguists, phoneticians, and speech scientists) who have worked in 43 countries and used 27 languages in professional practice. Seventeen panel members met for a 1-day workshop to identify key points for inclusion in the tutorial, 26 panel members contributed to writing this tutorial, and 34 members contributed to revising this tutorial online (some members contributed to more than 1 task).

**Results:**

This tutorial draws on international research evidence and professional expertise to provide a comprehensive overview of working with multilingual children with suspected speech sound disorders. This overview addresses referral, case history, assessment, analysis, diagnosis, and goal setting and the SLP's cultural competence and preparation for working with interpreters and multicultural support workers and dealing with organizational and government barriers to and facilitators of culturally competent practice.

**Conclusion:**

The issues raised in this tutorial are applied in a hypothetical case study of an English-speaking SLP's assessment of a multilingual Cantonese- and English-speaking 4-year-old boy. Resources are listed throughout the tutorial.

The development of children's multilingual competency in their home language(s) and the dominant language plays an important role in children's cultural identity, well-being, and sense of self (De Houwer, 2015; Puig, 2010). Speaking multiple languages may have academic benefits including enhanced cognitive skills (e.g., executive functioning and working memory) and social benefits including enhanced relationships (e.g., with grandparents) and participation in community activities (Adesope, Lavin, Thompson, & Ungerleider, 2010; Nguyen & Astington, 2014; Park & Sarkar, 2007). People in many parts of the world have a monolingual mindset (Hajek & Slaughter, 2014) that (mis)takes monolingualism for linguistic health and competence. In some instances, monolingualism is recommended as the “cure” for suspected or actual speech and language disorders (Cruz-Ferreira, 2011). However, recent large-scale longitudinal research has revealed that children speaking more than one language achieved educational and social-emotional outcomes similar to those of their monolingual peers (McLeod, Harrison, Whiteford, & Walker, 2016). In a systematic review, “limited evidence to suggest that bilingual children develop speech at a slower rate than their monolingual peers” was found (Hambly, Wren, McLeod, & Roulstone, 2013, p. 1).

Timing, amount, and quality of language exposure influence the level of proficiency in each of the languages a person speaks. Although simultaneous multilingual speakers are exposed to multiple languages from birth, sequential multilingual speakers establish their first language(s) in the home environment and then learn additional languages in educational or community contexts. Kohnert (2010) proposed a theoretical account of multilingual language development in which she identified three common characteristics of typical development featured among both simultaneous and sequential multilingual speakers: distributed skills and uneven ability, cross-language associations, and individual variation. Each of these characteristics has important implications for the assessment of multilingual children.

The common pattern of distributed skills and uneven ability is manifested by varying levels of proficiency in each of the child's languages. Language dominance is not a static construct and may vary depending on age, speaking partners, speaking contexts, and opportunities to develop certain skills in each language. Thus, an assessment of each language may show different strengths in particular language domains distributed unevenly across the languages (Kohnert & Bates, 2002), emphasizing the need to regularly assess all languages to gain an accurate picture of language capacities.

Cross-language associations among multilingual children are commonly known as cross-linguistic transfer. These effects can occur at the phonological, lexical–semantic, and morphosyntactic levels of language. Transfer effects between languages can be positive (facilitating language performance) or negative (impeding language performance), with the extent of transfer depending on how similar the languages are (Döpke, 2000). Transfer effects reveal that multiple languages are mediated through a central language processing mechanism even when they are functionally independent (Blumenfeld & Marian, 2009). The cognitive interaction of languages within this mechanism explains why the languages of multilingual speakers are impacted when speech sound disorders (SSD) and/or language impairment is present.

Considerable individual variation in multilingual language acquisition and skill development is due to linguistic, personal, and environmental factors. Thus, group norms for multilingual development are difficult to establish even when groups are tightly defined and may appear to be homogeneous (Pham & Kohnert, 2010). Therefore, the best practice for assessing multilingual children requires multiple measures to assess all languages at multiple points in time (Kohnert, 2013).

## Tutorial Overview and Aims

This tutorial provides guidance for speech-language pathologists (SLPs) assessing children's speech, particularly to differentiate children with SSD^1^ from those with speech differences.^2^ The overarching aim of this tutorial is to support SLPs' assessment of multilingual children^3^ with SSD, principally children who speak languages not shared with the SLP (known in other countries as fonoaudióloga/o, logopeda, logopedist, logoped, logopédiste, orthophoniste, patóloga/o de habla y lenguaje, speech pathologist, speech and language therapist, etc.).

The complexities of assessing multilingual children's speech have been discussed previously (Caesar & Kohler, 2007; Jordaan, 2008; Maul, 2015; Williams & McLeod, 2012) and include “referral, assessment, intervention, service delivery, cultural competence, knowledge of other languages, training, and collaboration with interpreters” (McLeod, Verdon, Bowen, & International Expert Panel on Multilingual Children's Speech [IEPMCS], 2013, p. 376). SLPs have acknowledged competence in the assessment of monolingual children's speech but must consider additional aspects when assessing the speech of children who speak nondominant languages and dialects.

The specific aims of this tutorial are as follows:

1. Provide guidelines for the assessment of multilingual children with suspected SSD who do not speak the same language(s) as the SLP
2. Identify key aspects and resources for the assessment of multilingual children with suspected SSD
3. Provide a case study to demonstrate the application of the principles within this tutorial

This tutorial is aspirational, grounded in both currently available empirical evidence and expert opinion for assessing multilingual children with suspected SSD.

## Development of This Tutorial

The IEPMCS formed in 2012 and has subsequently expanded, comprising people with specialist knowledge and publications in the field of SSD and multilingualism. A subset of the IEPMCS (17 members who had worked in 21 countries and spoke 16 languages) met in Stockholm, Sweden to consider current literature (e.g., Verdon, McLeod, & Wong, 2015a) and brainstorm best practices for assessment of multilingual children's speech. Major themes from the 50-page transcript of the 3-hr discussion were identified by the first two authors of this tutorial and served as a heading scaffold for its development. Twenty-six members of the IEPMCS wrote sections of the tutorial based on these headings, which was moderated by the two authors to create a draft, which was subsequently reviewed and revised by additional members of the IEPMCS. In total, 46 researchers (SLPs, linguists, phoneticians, and speech scientists) who had worked in 43 countries and used 27 languages in professional practice contributed to the development of this tutorial (see the Acknowledgments for a complete list of contributors). The recommendations of the IEPMCS for the assessment of multilingual children with suspected SSD follows.

## Speech Assessment of Multilingual Children

A key objective of speech assessment is to identify the presence or absence of SSD and typically includes referral, case history, assessment of speech production, additional areas of assessment (intelligibility, acceptability, stimulability, speech perception, phonological processing, language, hearing, oral structure and function, nonverbal intelligence, and participation), analysis, diagnosis, and goal setting.

### Referral for a Speech Assessment

The prevalence of SSD is similar in monolingual and multilingual populations (Hambly et al., 2013). However, multilingual children are at risk of being both over- and underreferred for speech-language pathology services and special education (MacSwan & Rolstad, 2006; Stow & Dodd, 2005). Multilingual children may appear to have patterns of speech and language acquisition that differ from those of monolingual children (e.g., delayed acquisition of consonant clusters because of transfer from the child's first language that does not use consonant clusters) and thus be overreferred for services. However, multilingual children with SSD may also fail to be referred for clinical assistance because communication difficulties (e.g., unintelligible speech) may be misinterpreted or misdiagnosed as features of multilingualism (Kritikos, 2003; Skahan, Watson, & Lof, 2007). Parents may also choose not to access services because of cultural beliefs (e.g., Bedford, Mackey, Parvin, Muhit, & Murthy, 2013; Berk, Schur, Chavez, & Frankel, 2000) or limited knowledge about speech-language pathology. SLPs should engage the multilingual child's community to discuss concerns and raise awareness of multilingual development (Cruz-Ferreira, 2010). Providing education about referral sources can potentially reduce over- and underreferral levels.

### Case History

Because no two multilingual children are alike, the SLP must obtain a thorough and culturally sensitive case history that takes into account the child's current and past cultural and linguistic environment (Shipley & McAfee, 2009). In addition to the information collected for monolingual children, such as child and family demographics, the family's needs and concerns, and the child's developmental milestones, the case history for the multilingual child also must include a comprehensive language profile. This profile should include the age at which the child was exposed to each language, the amount of exposure to and use of each language on a typical day, the people who speak each language to the child (e.g., parents, siblings, teacher, grandparents, or friends), the settings or context for language use (e.g., home, religious settings, community groups, or school), the child's preferred language (e.g., for music, dreams, counting, or thoughts), and the child's dominant languages (which may also vary as a function of partner, purpose, and context). Language dominance is not a static construct and changes as a function of time, age, task, and modality (Kohnert, 2013); therefore, language input, output, and proficiency should be recalculated each time a clinician performs an assessment. A comprehensive language profile is important because the child's proficiency in each language will be impacted by this variability and the information can be used to guide the interpretation of the assessment results, for setting intervention goals, and for developing contexts for generalization of new skills (Shipley & McAfee, 2009). Comprehensive language and developmental profiles can be obtained using the following:

- Alberta Language and Development Questionnaire (Paradis, Emmerzael, & Duncan, 2010; http://www.ualberta.ca/linguistics/cheslcentre/questionnaires#ALDeQ)
- Alberta Language Environment Questionnaire (Paradis, 2011; http://www.ualberta.ca/linguistics/cheslcentre/questionnaires#ALEQ)
- Bilingual English-Spanish Assessment Questionnaire (Peña, Gutiérrez-Clellen, Iglesias, Goldstein, & Bedore, 2014)
- Language Background Questionnaires (National Heritage Language Resource Center, 2015; http://web.international.ucla.edu/nhlrc/data/questionnaires)
- Zusatzmodul Anamnese bei zweisprachigen Kindern [Additional module for the case history of bilingual children] (Gumpert, 2014)

Additional information about the parents' language histories could be gathered using the Language History Questionnaire 2.0 (Li, Zhang, Tsai, & Puls, 2013; http://blclab.org/language-history-questionnaire/).

Knowledge of a family's worldview and cultural frame of reference is essential for providing speech-language pathology services that are meaningful and relevant for culturally and linguistically diverse families. Families may have multiple cultural influences upon their values, language preferences, kin structure, child rearing practices, religion, roles, responsibilities of family members (as caregivers, disciplinarians, socializers, and/or decision makers), perceptions of health, and behaviors across different domains of their life (McNamara, 2007). Cultural health and wellness practices may be based upon the family's explanatory models or the belief systems used by a cultural group to explain for health, illness, and disability (Hopf, McLeod, McDonagh, & Rakanace, in press; Kleinman, Eisenberg, & Good, 1978). For example, Kathard (1998) described a family who believed that their child's stuttering was a result either of the baby being left out in the rain or the parents' failure to inform their ancestors about the baby's imminent arrival. Families' diverse worldviews must be sought and treated with respect. It is also useful to gather information from others who influence the child's acquisition of communication skills (siblings, extended family, educators, and peers). Consideration of the cultural beliefs of families and communities also requires SLPs to reflect on their own cultural competence (discussed in detail later in this tutorial).

### Scope of Assessment

As in the monolingual context, formal and informal measures will be used to assess the multilingual child, considering the following: (a) speech production at the level of both single words and connected speech in each of the child's languages, (b) intelligibility and acceptability, (c) stimulability, (d) speech perception and phonological awareness, (e) hearing and oromotor structure and function, (f) language, (g) nonverbal intelligence, and (h) participation.

Assessments should target all of the child's languages (Gildersleeve-Neumann & Goldstein, 2012); therefore, the assessment will require broader data collection and more time than needed for monolingual children to yield an accurate diagnosis. There are several reasons why assessments should be conducted in all of the languages used by the child. First, languages differ in phonological and phonotactic structures: consonants, vowels, syllable types, word shapes, and suprasegmentals (e.g., Finnish has 13 consonants and Sesotho has 40). Second, multilingual children may not exhibit the same phonological skills in each language (Holm, Dodd, Stow, & Pert, 1999). Third, multilingual children's use (i.e., how frequently they hear and speak each language) and proficiency (i.e., how well they hear and speak each language) differ by language. Fourth, multilingual children's language history differs by language; they might begin to acquire each language at different time points, resulting in acquisition differences in each language. Assessment of all of the child's languages will aid in ensuring a reliable and valid diagnosis and an evidence-based link to the design of treatment goals, should intervention be necessary.

### Assessment of Speech Production

It is useful to observe the child's communication in a range of contexts and tasks. Single word tasks are the most efficient method for collecting data concerning the use of word shapes, prosody, and phonemes (consonants, vowels, and tones if appropriate) across word positions within each language. Connected speech samples provide information on the child's accuracy, intelligibility, and use of prosody.^4^ The procedures used to collect single word and connected speech samples in the multilingual context will depend upon the language backgrounds of the SLP, the language backgrounds and proficiency of the child, the availability of interpreters, and the availability of published tests in the languages spoken by the child. To undertake an assessment in another language, SLPs can take the following steps:

1. Familiarize themselves with the language and assessment tool or test.
2. Train a native speaker (interpreter or parent) to help administer the test.
3. Record a competent adult speaker (e.g., parent) or age-matched peer who speaks the same language and dialect undertaking the test. Their results can act as a comparative measure.
4. Audio- and video-record the child with high-quality recording equipment and microphones using uncompressed file formats (e.g., .wav; Vogel & Morgan, 2009).
5. Transcribe and analyze the child's speech using knowledge of phonetics and phonology to identify whether a need is present (remembering not to apply norms or standardized scoring based on monolingual children's speech).
6. Identify whether the child's speech is significantly different from the comparative measure.
7. Develop an intervention plan with the family and interpreter.

#### Formal Assessments in Languages Other Than English

Numerous speech assessments have been created to assess children who speak languages other than English. McLeod and Verdon (2014) reviewed 30 commercially available assessments in 19 languages other than English: Cantonese, Danish, Finnish, German, Greek, Japanese, Korean, Maltese-English, Norwegian, Pakistani-heritage languages (Mirpuri, Punjabi, and Urdu), Portuguese, Putonghua (Mandarin), Romanian, Slovenian, Spanish, Swedish, and Turkish. Many of these speech assessments were similar to those for English (e.g., presentation of stimuli and score forms), but some included larger normative samples and provided more extensive assessment and analysis. However, few of the 30 assessments were developed for or normed with multilingual populations, with the exception of tests for speakers of

- Maltese-English: Maltese-English Speech Assessment (Grech, Dodd, & Franklin, 2011)
- Pakistani heritage languages-English: Bilingual Speech Sound Screen: Pakistani Heritage Languages (Stow & Pert, 2006)
- Spanish-English: Bilingual English-Spanish Assessment (Peña et al., 2014); Contextual Probes of Articulation Competence: Spanish (Goldstein & Iglesias, 2006); Preschool Language Scales, 5th Edition, Spanish Edition: Articulation Screener (Zimmerman, Steiner, & Pond, 2012); Spanish Articulation Measures (Mattes, 1995); Spanish Language Assessment Procedures (Mattes, 1985); Spanish Preschool Articulation Test (Tsugawa, 2002)
- Turkish-German: Türkisch-Artikulations-Test [Turkish Articulation Test] (Nas, 2010)

In addition to the McLeod and Verdon review, a comprehensive list of assessments in languages other than English is available on the Multilingual Children's Speech website (http://www.csu.edu.au/research/multilingual-speech/speech-assessments) and includes assessments published as appendices in journal articles, on university websites, and other sources. Researchers from the University of British Columbia also have developed the International Phonological Development website (http://phonodevelopment.sites.olt.ubc.ca/) that contains free assessments in languages such as Arabic, Bulgarian, English, French, German, Icelandic, Japanese, Mandarin, Ojibwe, Portuguese, Punjabi, Slovenian, Spanish, Swedish, and Tagalog.

The content of each assessment tool must be critically evaluated from both a cultural and a linguistic perspective before the tool is used with multilingual children. Vocabulary items and pictures in the assessment tool may be unfamiliar to multilingual children raised outside that country or region. Adult target pronunciations presented in the tool may not be the same as those of the child's dialect. For example, Mandarin (Putonghua) as spoken in Beijing is phonologically different from equivalent standard languages in Taiwan, Malaysia, Hong Kong, and Singapore (Lee & Ballard, 2011). The SLP must be cautious when interpreting results from existing tools because most have been developed for monolingual populations using one (standard) dialect. Norms or standardized scoring based on monolingual populations cannot be used to draw conclusions about multilingual children's speech because these norms do not fully capture the competence of the multilingual individual's skills.

#### Adaptation of Assessments From One Language to Another

Adaptation of assessments from one language to another is not recommended, particularly for speech assessments, because phoneme inventories differ across languages. Pascoe and Norman (2011) suggested that starting from a blank page is more appropriate than adapting something developed for an entirely different population, place, and language. Use of assessments in the dominant language comes with risks: (a) overidentification of difficulties, such as classification of “mispronunciation” as SSD because of linguistic transfer; (b) exclusion of phonemes from the nondominant languages spoken by the child; (c) omission of particular diagnostic markers; and (d) confusion of or offense to clients through the use of culturally insensitive methods and materials (Grech & Dodd, 2007; Hack, Marinova-Todd, & Bernhardt, 2012).

#### Informal Assessments in Languages Other Than English

When formal assessment protocols are unavailable or inappropriate, the SLP may choose to develop informal assessments to obtain a speech sample using single word naming. To develop such an assessment, the SLP requires knowledge of the consonant, vowel, word, and prosodic structure inventories of the language to evaluate the child's phonological systems. The SLP also must determine whether the child's speech is typical for his or her age, whether by inventory development or error patterns.

SLPs can identify the phonetic and word structure inventories of languages by drawing on available resources:

- Speech Accent Archive (http://accent.gmu.edu/browse.php)
- Multilingual Children's Speech (http://www.csu.edu.au/research/multilingual-speech/languages)
- International Phonetic Association Handbook (1999) (https://www.internationalphoneticassociation.org/content/ipa-handbook-downloads)
- Cross-Linguistic Phonology Project (https://www.phonodevelopment.sites.olt.ubc.ca)

The SLP can then work with a native speaker to choose a set of culturally suitable screening words that contain all target phonemes and major word structures and can select culturally sensitive pictures to elicit words. The SLP can also work with parents and interpreters to develop a more in-depth set of words to follow up on particular areas of difficulty. Computer technology may also be used to assist with speech assessment in multiple languages (Schaefer, Bowyer-Crane, Herrmann, & Fricke, 2016). Further information for test development has been published by McLeod (2012) and Bérubé, Bernhardt, and Stemberger (2013).

#### Transcription of Speech

During their training, SLPs tend to develop transcription proficiency in their native language but may have limited skill in transcribing languages that they cannot speak. Working with children from multilingual environments necessitates the maintenance and extension of SLPs' phonetic and phonemic transcription skills. The International Phonetic Alphabet (IPA; International Phonetic Association, 2015) enables access to information on the place and manner of articulation of all sounds used in the world's languages. The UK and Ireland Specialists in Specific Speech Impairment Network (2013) published guidelines for the transcription of children's speech. This document supported the need for confidence in “using the full set of IPA and, if necessary, extIPA symbols when treating children with structural/neurological or hearing impairment or non-native English speakers, where phonetic-level variation from standard speech is likely” (p. 3). The guidelines acknowledged that narrow phonetic transcription was not always necessary but was more likely to be required when assessing children from multilingual backgrounds because of allophonic variation in phoneme production across languages. Without using narrow phonetic transcription, the SLP may assume that a child is presenting with distortions when in fact the child is producing the phoneme in a language-specific manner.

With practice, SLPs can develop skills to transcribe children's speech even in languages that they do not understand. Several excellent websites have sound files that SLPs can access to improve their ability to perceive unfamiliar consonants and vowels (e.g., Eric Armstrong at York University: http://www.yorku.ca/earmstro/ipa/index.html; University of Glasgow's Seeing Speech project: http://www.seeingspeech.ac.uk, Lawson et al., 2015). Phonetic transcription of an unknown language encourages SLPs to listen to what the child is producing without the influence of what the child “should” be saying. Transcription of new sounds requires practice and feedback from a native speaker. Caution is needed, however, because native speakers can also be influenced by orthography. For example, English speakers might say that the same sound occurs at the beginning and end of the word “sounds,” when in fact, the first speech sound is /s/ and the end sound is /z/. Lockart and McLeod (2013) found that English-speaking SLP students had skills to identify errors and transcribe Cantonese children's consonants in a single word task, and transcription accuracy was increased when these student SLPs were able to hear a Cantonese-adult model of the words and were provided with information about Cantonese phonology. Transcription is significantly easier to undertake with single word samples than with connected speech. Transcribing a spontaneous speech sample in an unfamiliar language can be challenging, because SLPs need to segment words from continuous speech and may not know where words begin and end. In this instance, assistance from a native speaker may be required.

#### Intelligibility and Acceptability

Intelligibility—the degree to which a child's speech is understood—can be screened using the Intelligibility in Context Scale (McLeod, Harrison, & McCormack, 2012a), which is available for free in 60 languages from the Multilingual Children's Speech website (http://www.csu.edu.au/research/multilingual-speech/ics), validated in several languages (e.g., Cantonese: Ng, To, & McLeod, 2014; Jamaican Creole: Washington, McDonald, McLeod, Crowe, & Devonish, in press), and normed in English for monolingual and multilingual children (McLeod, Crowe, & Shahaeian, 2015). Speech intelligibility assessments also are available in a range of languages (e.g., Swedish Test of Intelligibility for Children: Lagerberg et al., 2015).

Speech acceptability is also an important consideration for multilingual children. This construct differs from speech intelligibility and can include transfer from one language to another that may not be considered to be socially acceptable in some contexts. For example, speakers of some languages and dialects (e.g., Australian Aboriginal English, Pacific Island English, and Tongan) do not use the voiced and voiceless dental fricatives /θ, ð/ and may produce *think* /θɪŋk/ as *fink* [fɪŋk]. Some Standard English speakers do not consider this pronunciation acceptable, even though the speech is intelligible. Speech acceptability may also be influenced by transfer of features of prosody and resonance.

#### Stimulability

*Stimulability* refers to a child's ability to accurately imitate a modeled phoneme (Miccio, 2002). Stimulability is usually assessed by asking the child to look at the SLP's face (or in the multilingual context, the face of a family member), listen to a sound, and repeat the same sound (Lof, 1996). Repetition of single words can be a useful means of supplementing the phonetic inventory with sounds within the child's ability but not within their repertoire. Repetition of single words can be useful for assessing stimulability for the full range of sounds across all of the languages of the child. Stimulability can also be assessed through dynamic assessment (described later in this tutorial) to determine the level (isolation, syllables, words, etc.) and range of cues needed for a child to be stimulable (Glaspey & Stoel-Gammon, 2007).

#### Speech Perception and Phonological Processing

In addition to speech production data, assessment of speech processing should also be carried out. To assess a child's speech perception skills, the SLP can use an identification of mispronunciations task, such as those described by McNeill and Hesketh (2010) and Rvachew and Grawburg (2006). Phonological processing can also be assessed using nonword repetition (NWR) tasks. NWR task accuracy can be influenced by the languages and dialects spoken by the child (Windsor, Kohnert, Lobitz, & Pham, 2010); consequently, the SLP may consider the Syllable Repetition Task (Shriberg et al., 2009), which contains only four consonants and one vowel commonly found across languages, keeping in mind that the reference data provided for English speakers will not be valid for children who speak other languages. Numerous other NWR tasks are available in languages other than English (e.g., Turkish Nonword Repetition Test; Topbaş, Kaçar-Kütükçü, & Kopkalli-Yavuz, 2014). Chiat (2015) provided a review of NWR tasks in languages other than English.

### Additional Areas of Assessment

#### Language Skills

Assessing multilingual children's receptive and expressive vocabulary, morphology, syntax, and discourse skills in languages that are not spoken by the SLP poses similar challenges to the assessment of multilingual children's speech. When a narrative sample or play sample is being collected for the language assessment, that sample can double as a connected speech sample. A range of bilingual assessments for children with specific language impairment has been developed by SLPs in the European Union as part of the COST Action Project IS0804 (http://bi-sli.org/).

#### Hearing and Oral Structure and Function

Multilingual children may have missed routine developmental and medical screening if they were born in resource-constrained countries or did not access these services because of the timing of relocation. To rule out hearing loss as a cause or contributing factor to SSD, children should have their hearing tested prior to receiving a speech assessment. An examination of oral structure and function should also be conducted to identify possible underlying causes of SSD, including observation of facial characteristics, dentition, tongue, palatal, and pharyngeal areas and maximum performance tasks (e.g., Robbins & Klee, 1987; Rvachew & Brosseau-Lapré, 2012).

#### Nonverbal Intelligence

Tests of nonverbal intelligence, typically administered by psychologists, can be useful for helping to determine developmental profiles. The Primary Test of Nonverbal Intelligence (Ehrler & McGhee, 2008) is one test that the SLP can administer and is available for speakers of nine languages (including English). This test has a point-response format and provides a brief norm-referenced evaluation of nonverbal reasoning skills in children.

#### Participation

Although traditional speech assessments are focused on discrete skills (e.g., speech sound production), the goal of intervention is to increase children's ability to use speech to participate in their environment (Pennington, 2010). The International Classification of Functioning, Disability and Health: Children and Youth Version (World Health Organization, 2007) provides a structure for examining children's activities, participation, and real-world functioning and is the basis for the Focus on the Outcomes of Communication Under Six (FOCUS; Thomas-Stonell, Washington, Oddson, Robertson, & Rosenbaum, 2013), which is an easily accessible, SLP- and parent-friendly tool. The FOCUS can be used to measure the child's communicative participation in their environment and to monitor the impact of intervention, in this context on the basis of the child's participation in diverse cultural and linguistic environments. The FOCUS has been translated into various languages (e.g., German: Neumann, Salm, Rietz, & Stenneken, 2017; and French: Pominville, Turcotte, Oddson, Rosenbaum, & Thomas-Stonell, 2015).

#### Strengths-Based Assessment

Multilingual families and communities have diverse views on childhood and disability, necessitating consideration of strengths and assets and areas of difficulty and difference. In a strengths-based or asset-based approach to assessment, parents are invited to share what is special about their child and offer insights into their world and the child's place within it. Louw (2009) detailed a conversation between a family and therapist that was started to explore both the strengths and needs of a child with communication difficulties: “[T]he interview began with Sophie [the mother] sharing what was special about Tumi [the child] and her expectations of the assessment, without having her explain ‘problems’ … this was a ‘family conversation’ … assisted by an experienced interpreter engaged to lessen barriers of language and culture” (p. 170). A strengths-based approach to assessment enables SLPs to draw on the child's areas of skill when designing intervention goals, for example, using established word and syllable structures when targeting new phonemes (Bernhardt & Stemberger, 2000).

#### Dynamic Assessment

Conducting assessments with children who are culturally and linguistically diverse can be complex, requiring an approach that reduces possible biases and inaccuracies in reporting (Lidz & Peña, 1996). The use of dynamic assessment with multilingual children has been identified as a culturally sensitive approach because it supports the observation of performance and competence regardless of the language(s) spoken. Using a dynamic assessment approach, the SLP can (a) establish baseline functioning, (b) identify appropriate targets and contexts for intervention, and (c) measure change (Peña, 2000). The child is observed in structured and unstructured settings, providing a way to identify the child's skills, learning potential, and learning process and to determine potentially effective methods of teaching (Lidz, 1991).

One type of dynamic assessment includes a pretest–intervene–posttest format in which a series of tasks are presented, taught, and then evaluated within a mediated learning experience (Westby, Stevens Dominguez, & Oetter, 1996). Using dynamic assessment, the SLP manipulates the testing situation so that optimal observation can occur, with children encouraged to talk about what they are thinking. Three types of dynamic assessment are useful for considering the speech of multilingual children:

- Dynamic Assessment of Preschoolers' Proficiency in Learning English (Hasson, Camilleri, Jones, Smith, & Dodd, 2013)
- Glaspey Dynamic Assessment of Phonology (Glaspey & MacLeod, 2010)
- Dynamic Assessment of Phonological Awareness for Children with SSD (Gillam & Ford, 2012)

### Analysis of Speech Production

#### Independent and Relational Analysis

An independent analysis describes the child's phonological system independently of the adult system (Stoel-Gammon & Dunn, 1985) and includes two key areas. The phonetic repertoire is the inventory of the phones produced by the child, whether correctly or not and whether or not the phone is typically used in the child's language(s). For each of the child's languages, consonants are organized by place and manner of articulation, whereas vowels are organized according to the location of the tongue (front, central, or back) and degree of openness (low, mid, or high). Phones produced only once by the child are typically shown in parentheses. The phonemic repertoire is the inventory of the phonemes that the child uses to contrast meaning. An independent analysis is particularly useful for analyzing the speech of multilingual children because it allows for a summary of children's phonetic repertoire without applying language-based restrictions on the sounds included in the analysis.

A relational analysis compares the child's production to the adult target (Stoel-Gammon & Dunn, 1985). With the substitution, omission, deletion, and addition analysis method, each phoneme is judged as correct, a substitution, an omission, a distortion, or an addition (Shriberg & Kent, 2003; Van Riper, 1939). When conducting a substitution error analysis with multilingual children, the SLP should account for dialectal differences by giving the child credit for a “mispronunciation” when that pronunciation could be the result of dialectal differences or cross-linguistic transfer (Toohill, McLeod, & McCormack, 2012; Velleman & Pearson, 2010). For example, for a child that speaks French as her or his first language and English as the second language, the production of [f] for /θ/ (European French) or [t] for /θ/ (Canadian French) would be indicative of the cross-linguistic influence of French upon the child's production of English rather than the presence of SSD because /θ/ is not present in French. Relational analyses also include calculations of the percentage of consonants correct (PCC; Shriberg, Austin, Lewis, McSweeny, & Wilson, 1997), and this metric has been applied to the speech of multilingual children (e.g., Fabiano-Smith & Goldstein, 2010). Phonological processes (patterns) such as final consonant deletion and cluster reduction can also be included in a relational analysis. However, typical processes differ among languages. For example, backing may be relatively uncommon in English but more common in Cantonese (To, Cheung, & McLeod, 2013). Goldstein and Fabiano (2007) described how to complete relational analyses for multilingual children.

#### Family Member Contrastive Analysis

When the SLP is not familiar with a child's language(s), the child's family members can provide assistance because they have similar linguistic influences and can model target productions (McGregor, Williams, Hearst, & Johnson, 1997). In the absence of an interpreter, the SLP can record a child's single word productions (preferably at least twice) and then record the family member's productions of the same words. Subsequently, the SLP can (a) transcribe and compare the family member's and the child's productions to identify phonetic differences, (b) use acoustic analysis to compare the child's versus family member's productions, and/or (c) ask the family member to identify correctly produced words and then calculate the proportion of whole words correct (Ingram & Ingram, 2001). To avoid syntactic and morphological interference (i.e., the parent says that the production is incorrect because of a morphological rather than phonological error), it is best to use only noun stems as target words. A combination of these methods can help the SLP analyze and evaluate the child's phonological status and decide whether intervention is needed.

#### Nonlinear Analysis

Constraint-based nonlinear phonological frameworks have served as a basis for phonological intervention planning for English since 1990 (Bernhardt, 1990, 1992; Bernhardt & Stemberger, 1998). A fundamental concept is that phonology “isnotjustastreamofconsecutivespeechsounds” but rather is a hierarchy of phonological levels from the prosodic phrase to phonological features, with intervening levels such as foot, syllable, onset, rime, and phoneme. The various levels are both autonomous, showing their own patterns, and interactive, with influences from one level to another (e.g., restriction of features by syllable position). Nonlinear phonological intervention exploits strengths (faithfulness) at one level of the hierarchy to address needs (markedness) at others. Barlow and colleagues have applied aspects of constraint-based analysis drawing on theoretical ideas from optimality theory for analyzing Spanish (Barlow, 2005; Barlow & Enríquez, 2007; Fabiano-Smith & Barlow, 2010) and Vietnamese (Tang & Barlow, 2006). A constraint-based analysis form is available for 14 languages on the International Phonological Development website (http://phonodevelopment.sites.olt.ubc.ca/) to assist with systematic analysis of the multiple levels (e.g., Bernhardt & Zhao, 2010; Bérubé et al., 2013; Chen, Bernhardt & Stemberger, 2016). Online tutorials in English, French, and Spanish are also provided on the website with demonstrations of how to conduct a nonlinear analysis.

#### Instrumental and Acoustic Analysis

Instrumental assessment and analysis using electropalatography and ultrasound provide information on tongue movement and contact patterns during speech (Bernhardt, Stemberger, & Bacsfalvi, 2010; Gibbon, 2008; Hardcastle & Gibbon, 2005; Zharkova, Gibbon, & Hardcastle, 2015). Acoustic analysis of recorded speech samples provides objective information about voicing, vowel and sibilant quality, epenthesis (vowel or consonant insertion), pitch, prosody, and rhythm. Speech analysis programs are commercially available, some of which are free and easily accessible for use on personal computers, including Praat (Boersma & Weenink, 2014; http://www.fon.hum.uva.nl/praat/) and Phon (Rose & MacWhinney, 2014; http://childes.psy.cmu.edu/phon/). Other tutorials have been developed specifically for SLPs and audiologists to provide guidance for working with sound files and conducting a variety of computerized speech and language analyses (e.g., Ingram, Bunta, & Ingram, 2004; Price, Hendricks, & Cook, 2010). When working with multilingual children, acoustic analysis is useful for assessing and tracking client progress (Ingram et al., 2004; MacLeod & Glaspey, 2014) and can reveal unique information not easily available via other means (Li, Edwards, & Beckman, 2009). Voice onset time (VOT) has been studied to differentiate between voiced and voiceless plosives (stops) in multilingual populations (Lee & Iverson, 2011). In various VOT studies, multilingual speakers performed differently from their monolingual peers, although the target language VOTs could still be differentiated, depending on age of acquisition and proficiency levels (Fabiano-Smith & Bunta, 2012; MacLeod & Stoel-Gammon, 2005). Acoustic analyses are also useful for looking at vowels (e.g., the English low front vowel: Bunta & Norton, 2012; and Korean–English vowels: Lee & Iverson, 2012) or fricatives and affricates (e.g., Glaspey & MacLeod, 2010). Acoustic analyses also are helpful for investigating the phonological patterns of multilingual children with speech, language, or hearing disorders, including children who use cochlear implants (Bunta, 2014). Thus, practicing SLPs can benefit from selective application of acoustic analyses to assessment and analysis.

### Diagnosis and Goal Setting

The diagnosis of SSD and development of consequent management plans entail (a) identification of areas of strength and potential difficulties; (b) establishment of baselines across communication domains; and (c) selection of intervention goals, strategies, and management options. The child's aptitude in and motivation for speaking each of her or his languages plays a decisive role in choosing goals and objectives. Differentiation of SSD from difficulties related to learning an additional language is crucial (McGregor et al., 1997). Children's speech performance should ideally be compared with age-matched data of typically developing children acquiring the same language(s). The lack of appropriate norms for multilingual children is an acute problem, given that the SLP should assess speech and language performance in all of the languages a child speaks. When a child has difficulty in only one language, the difficulty typically results from cross-linguistic transfer or lack of exposure to that language (Kohnert, Yim, Nett, Kan, & Duran, 2005; Paradis, Genesee, & Crago, 2011). When local norms for multilingual children are not available, caregivers or interpreters (among others) may be able to indicate whether the speech production patterns observed in the dominant language occur in the home language(s) of age-matched peers or adults. When patterns indicate a (dialectal) difference from standard production but not SSD, the question remains whether intervention is warranted. Accent modification or intelligibility enhancement may be of interest to some families who wish to improve speech intelligibility but may be excluded from the scope of practice in some services (American Speech-Language-Hearing Association, n.d.). However, SSD that is present but left untreated because of misdiagnosis as a speech difference or accent can have a lifelong impact upon children's social, academic, and occupational outcomes (Law, Garrett, Nye, & Dennis, 2003; McCormack, McLeod, McAllister, & Harrison, 2009).

It is important to consider how family and cultural attitudes affect acceptance and interpretation of diagnoses and disorder labels because values, priorities, and cultural acceptance of difference vary dramatically. Some cultures have a narrower range of variation for acceptable speech, resulting in higher rates of identification of and intervention for children with SSD. Thus, what one group considers mild SSD may be viewed as acceptable typical variation in another. Individuals and cultures also differ in their primary framework or explanatory models for understanding disability, and that framework can affect how readily a diagnosis is accepted, can create feelings of shame and guilt, and can affect what families view as the most suitable approaches for remediating difficulties (e.g., Kalyanpur & Harry, 2012). SLPs must listen to the family's perspective on the disability when deciding how best to share diagnostic results and determining a management plan, which may entail either doing nothing or setting up intervention goals.

SLPs generally recognize the importance of parental involvement in intervention for children with speech and language difficulties (Watts Pappas, McLeod, McAllister, & McKinnon, 2008). This approach is in accordance with legislatures supporting the rights of the child across the world, which has increased the involvement of parents in their children's interventions (Bowen & Cupples, 2004) including (a) working toward goals in the home or school language, (b) increasing the child's motivation for intervention, and (c) providing encouragement and opportunities to practice communication in a natural setting. Under certain conditions, parental support is vital, for instance, when practicing established, stimulable speech sounds and assisting children at the level of speech sound generalization. For multilingual children, parents can provide additional support for practicing new sounds in their home languages. However, cultural conventions (e.g., interaction styles and family structure) must be respected and used to inform goal setting (Hwa-Froelich & Vigil, 2004; Lee & Ballard, 2011). For example, Lee and Ballard (2011) noted that when working with more traditional Chinese parents, the expectation was a medical model of intervention, where the SLP takes a direct approach to “fix” the problem with little involvement from the parents. In other cultures, children and adults do not routinely interact with each other, and caregiving may be the responsibility of older siblings. As a consequence, children may be nonverbal in front of adult authority figures, and parents may feel uncomfortable in play activities with their children (Ballard & Farao, 2010). SLPs must gain this cultural knowledge when working with parents to negotiate communicative strategies, goals, and interventions for children.

#### Encouraging Communication in All Languages

When trying to provide the most effective and efficient speech and language services for multilingual children with SSD, SLPs and parents are understandably concerned about whether supporting more than one language is the best approach. Mounting evidence supports the use of all languages for multilingual children with speech, language, or hearing disorders when their families favor multilingualism (Guiberson, 2014; Kohnert et al., 2005; McConkey Robbins, 2007). Thus, when there is support from the family for the home language(s), encouraging the use of more communication (regardless of the language) can yield positive results for multilingual children with communication disorders. Intervention provided in all languages also can produce positive results (Kohnert et al., 2005; Thordardottir, Cloutier, Ménard, Pelland-Blais, & Rvachew, 2015), and generalization of speech goals has been noted across languages for children with SSD (Gildersleeve-Neumann & Goldstein, 2015).

### Professionals, Policy, and Workplaces

#### SLPs' Context and Cultural Competence

When working across cultures, people bring their own unique cultural lenses and understandings to a situation, which can lead to misunderstandings or power imbalances when each person's culture is not understood, acknowledged, and valued. To engage effectively in cross-cultural practice, SLPs must consider the impact of their own languages, cultures, and beliefs and the languages, cultures, and beliefs of the families they work with for guiding thinking, decisions, and actions (Spector, 1985; Verdon, McLeod, & Wong, 2015b). People's identities are influenced by their multiple languages and cultures, which can differ greatly from practices found in monolingual westernized cultures.

To bridge the cultural gap between SLPs and the families they work with and to provide a culturally safe environment for families, SLPs must first reflect on their own identities, becoming aware of their languages and cultures and how these attributes shape thinking, understanding, and approaches to practice (American Speech-Language-Hearing Association, 2004; Verdon et al., 2015b). The SLP can ask questions such as: What is my cultural background? What influences and informs my thinking? What values do I cherish? Do I have any biases? How might those values and backgrounds affect my work? Resources to support professional self-reflection are available from the American Speech-Language-Hearing Association (http://www.asha.org/uploadedFiles/Cultural-Competence-Checklist-Personal-Reflection.pdf).

To work competently across cultures, it is not essential to know many languages and dialects; rather, the SLP must be aware of the great diversity among families' languages, cultures, and identities and how this diversity can affect interactions in practice. Cultural competence requires a basic level of understanding of the client's culture (acknowledging individual differences between families) in addition to the features and structures of the client's language(s) and dialect(s) (i.e., phonology, semantics, morphology, syntax, and pragmatics). An understanding of the impact of culture on communication (e.g., eye contact, interpersonal proximity, and who speaks to whom) is also necessary. In some cultures, parents and children may be uncomfortable with direct questioning. For example, Chinese children may not respond to a question if they do not know the correct answer (To, 2016). In other cultural groups, children may behave differently in the company of adults. For example, Samoan children use different registers (formal or colloquial) in different speaking situations, and these registers contain different consonants (Ballard & Farao, 2008).

SLPs also must be aware of power imbalances that can arise from perceived differences in language or dialect status in certain cultural groups. The use of a high status or dominant language or dialect (such as standard English) can make people conscious of speaking a lower status language or dialect and could be seen as an expression of exclusion or exercising the global power of western cultural dominance over minority cultures (Simone, 1977). The SLP also may be perceived as holding power, given their educational accomplishments and occupation.

Research with SLPs engaging in practice with First Nations communities in Canada revealed strategies for culturally competent practice (Bernhardt, 2015).

- Find people in the community to guide you. Spend time with them and learn by working alongside them. Join in with public community events and accept invitations to events from community members that have nothing to do with speech or language but everything to do with becoming known and familiar in the community.
- Dress like other helpers in the community.
- Use plain English and learn useful phrases in the languages of the community.
- Learn how the community works with the concept of time. Do they want scheduled appointments or open days for appointments (i.e., first come–first served)?

SLPs must receive pre- and in-service training to develop knowledge of cultural diversity, language acquisition and use by multilingual people, and skills in supporting multiple languages that empower SLPs to work cross-linguistically.

#### Working With Interpreters and Multicultural Support Workers

When the SLP does not speak (all) the child's languages and dialects, a mediator who is familiar with those languages or dialects is needed. Family members can assist, but professional interpreters enhance objectivity and may make it easier to convey complex and specific information (Langdon & Saenz, 2015; Mettey, 2013). Interpreters may not be trained to assist in the administration of formal or standardized testing or to give an opinion about speech intelligibility (Isaac, 2005; Roger & Code, 2011). To undertake these tasks, training is needed for both the SLPs and the interpreter or mediator to ensure that exchanges attain the desired outcome. When organizing formal training for interpreters, Blumenthal (2007) recommends that the learning outcomes include knowledge about children's speech and language development, speech and language impairments, multidisciplinary diagnostic processes, testing protocols, and transcription of grammatical and phonological features. One recommended method for optimizing exchanges between SLPs and interpreters is to use the briefing–interaction–debriefing model (Langdon & Cheng, 2002):

- Briefing: SLP and interpreter meet before sessions to discuss assessment and intervention goals and make interpretation decisions.
- Interaction: SLP and interpreter work together with the child.
- Debriefing: SLP and interpreter review session outcomes and make follow-up plans.

During the interpreted sessions, both the interpreter and the SLP must watch the client (child and family). The SLP must stay alert for nonverbal cues, even when the language of the client is not understood. The responsibility of the assessment always stays with the SLP; the interpreter's role is to provide support. Additional guidance is available on the American Speech-Language-Hearing Association website (http://www.asha.org/practice/multicultural/issues/interpret.htm).

#### Policy Barriers and Facilitators for Culturally Competent Practice

Many of the strategies outlined above require SLPs to work outside of dominant models of practice, taking extra time to build relationships with families and communities from different cultural backgrounds. Some policies can act as facilitators for or barriers to engaging in culturally safe and appropriate practices by dictating the languages that can be used in practice and specifying assessments that must be undertaken for diagnosis. For example, in the United States, support of Spanish–English bilingual language acquisition in schools is prohibited in the state of Arizona under state educational policy. Therefore, SLPs are required to focus on English and are unable to provide assessment and intervention in the children's home language (Verdon et al., 2015b). In Germany, multilingual children are assessed on German language competence (Sprachstandserhebungsverfahren) 1 year before school entry. Children who fail these tests must attend German language training. Few multilingual kindergartens exist (2% of all German kindergartens; Frühe Mehrsprachigkeit und Kitas und Grundschulen, 2015), and few speech-language pathology services are provided in languages other than German (Chilla, Rothweiler, & Babur, 2013). Consequently, culturally competent practice for supporting multilingual children's speech acquisition is impacted by professional and government policies, including provision of increased time and resources (IEPMCS, 2012).

### Case Study

To draw together the recommendations of this tutorial, we conclude with a case study of a hypothetical boy called Tom who is aged 4;11 (years;months), speaks Cantonese and English, and lives in a large city in an English-speaking country.

#### SLP's Cultural Competence and Preparation

Carol is a monolingual English-speaking SLP who has limited experiences with other cultures. In her clinic, she has seen a few children who speak Spanish but has not had any referrals for children who speak other languages. In anticipation of Tom's assessment, she asked a Chinese neighbor to include her in events in the local Chinese community and provide information about Chinese culture. She looked up the consonant and vowel inventory of Cantonese in multiple sources, specifically the Speech Accent Archive (http://accent.gmu.edu), the Multilingual Children's Speech website (http://www.csu.edu.au/research/multilingual-speech/languages), the *Handbook of the International Phonetic Association* (Zee, 1999), and the *International Guide to Speech Acquisition* (So, 2007). Carol found that in Cantonese there are many more initial consonants (/p, p^h^, t, t^h^, k ,k^h^, k^w^, k^wh^, ts, ts^h^, f, s, h, w, j, l, m, n, ŋ/) than final consonants (/p, t, k, m, n, ŋ/), although these sounds mostly overlap with English articulations.^5^ She also learned that Cantonese has eight vowels, 11 diphthongs, and nine tones, and the most common syllable shape is CV. She investigated the age of acquisition of consonants, vowels, and tones and typical processes for Cantonese children in the *International Guide to Speech Acquisition* (So, 2007) and the population study by To et al. (2013). She learned that monolingual Cantonese children rarely have difficulty producing tones, which are typically acquired by 2;6 (To et al., 2013). She read some literature (e.g., Hu, Torr, & Whiteman, 2014; Verdon et al., 2015b) suggesting that some families may choose to prioritize English over maintenance of Cantonese, despite evidence highlighting possible benefits of multilingualism. She also read information on Chinese child-rearing practices and culture (e.g., Lee & Ballard, 2011; To, 2016, especially Table 6.5, pp. 143–144) indicating that testing is important to many Chinese families, children tend to be obedient, play is not highly valued as an educational method, parents learn through observation but are less likely to participate in sessions, and teachers (including SLPs) are seen as figures of authority. Carol was mindful that she would be working with an individual Chinese family, and although some cultural patterns will apply to most families, cultural stereotypes should not be relied on as a framework for assessment.

#### Referral: Who Thinks This Child Needs Help With His Speech?

Tom is due to begin English-speaking elementary school soon, and his parents and early childhood educators are concerned that others will not understand him at school. Tom's family indicated that they did not need an interpreter to provide case history information.

#### Case History: What Information Should Be Learned?

Carol interviewed the parents and learned that Tom lives with his parents and grandmother. They migrated to the United States from Hong Kong when Tom was a baby. The primary language spoken at home is Cantonese, and Tom has attended an early childhood center since age 3, where most educators speak English but some also speak Cantonese. Tom's parents are bilingual in Cantonese and English, and his grandmother speaks only Cantonese. Tom has been exposed to Cantonese since birth and began speaking English when he started going to the early childhood center. Carol administered the Bilingual Input-Output Survey (BIOS; Peña et al., 2014) as part of the case history (adapted by considering Cantonese instead of Spanish). Tom's BIOS score summary was as follows: language at home, 90% Cantonese input, 10% English input, 70% Cantonese output, 30% English output; language at school, 10% Cantonese input, 90% English input, 10% Cantonese output, 90% English output. If Tom is subsequently seen by Carol for ongoing management, she will repeat the BIOS regularly because language dominance is not considered a static construct (Kohnert, 2010).

There is no family history of speech and language difficulties. To gather a comprehensive language and developmental profile, Tom's parents completed the Alberta Language and Development Questionnaire (Paradis et al., 2010). Carol asked the family to describe what was special about Tom and to offer insights into their aspirations for him. They also discussed the family's understanding of Carol's role, their concerns, and what they hoped to achieve by attending speech-language pathology sessions.

#### Assessment: How Should Information Be Collected About This Child's Speech?

*English.* Carol undertook screening assessments of hearing and oral structure and function, and results were within normal limits. Because Tom spoke both English and Cantonese, Carol conducted a single-word, connected speech, and nonword assessment in English.

*Cantonese.* Tom's parents reported that Tom did not have difficulties using tones or vowels in Cantonese, but he had difficulty producing some Cantonese consonants. Carol asked Tom's parents to complete the Traditional Chinese version of the Intelligibility in Context Scale (McLeod, Harrison, & McCormack, 2012b, trans. by To & Ng), and they indicated that Tom was “sometimes” understood by family and friends (*M* = 3.1 [of a possible 5]). This score was lower than that for typically developing monolingual Cantonese children (*M* = 4.56, *SD* = 0.48) and for monolingual Cantonese children with SSD (*M* = 4.14, *SD* = 0.65; Ng et al., 2014). Carol considered contacting but could not locate an SLP who spoke Cantonese and English (e.g., via Skype with an SLP in Hong Kong) to assess Tom's Cantonese speech production. Carol then considered the list of assessments in languages other than English on the Multilingual Children's Speech website (http://www.csu.edu.au/research/multilingual-speech/speech-assessments) and in the article by McLeod and Verdon (2014). She found that the word list for the Cantonese Segmental Phonology Test (So, 1993) was available as an appendix in the article by So and Leung (2004). She also worked with a local Cantonese interpreter to determine that the word list was relevant for Cantonese-speaking children in her city and to source relevant pictures for each word. Carol and the interpreter conducted the Cantonese Segmental Phonology Test together, and Tom's speech was audio-recorded and transcribed in real time using the IPA. Carol also audio-recorded Tom's mother producing the test stimuli. Carol found out that English-speaking SLPs are able to transcribe the following Cantonese consonants with at least 70% accuracy: /m, n, f, s, h, j, w, l/ in initial position and /p, t, k, m, n, ŋ/ in final position (Lockart & McLeod, 2013). For the other consonants, she asked the interpreter and the parents whether the production was correct and checked online IPA websites to listen to the target productions.

#### Analysis: How Should the Data Be Interpreted?

Carol read that when children have a speech and/or language disorder, it occurs in all languages spoken by the child (Paradis et al., 2011). Tom's response on the NWR Syllable Repetition Task (Shriberg et al., 2009) was within normal limits for an English-speaking child. Tom's PCC in English was 63% (equivalent to “moderate-severe involvement”; Shriberg, Kwiatkowski, Best, Hengst, & Terselic-Weber, 1986). Carol recognized that his PCC was lower than expected for his age. To make sure that this low score was not due to a lack of exposure in English or to cross-linguistic transfer, she compared it to normative data from Cantonese–English bilingual children with exposure to both languages similar to that experienced by Tom (Dodd, Holm, & Wei, 1997). Carol found that the mean PCC in English of 16 Cantonese–English bilingual children 25–52 months of age with typical development was about 95%. In other research, the PCC in English of two Cantonese–English bilingual children 37 months of age with typical development was 75%–80% (Holm & Dodd, 1999). Carol noticed that on the English speech assessment Tom demonstrated final consonant deletion, initial consonant deletion, cluster reduction, backing, and stopping more than 40% of the time. She also noticed that on the Cantonese speech assessment he had deleted initial and final consonants and seemed to have difficulty producing stops and fricatives. In discussion with the interpreter and his parents, she concluded the following.

*Initial consonants.* Tom deleted initial consonants. Monolingual English-speaking and Cantonese-speaking children typically do not delete initial consonants. However, Holm and Dodd (2006) reported that initial consonant deletion was used by approximately 20% of 56 Cantonese–English-speaking children.

*Final consonants.* Young monolingual English-speaking and Cantonese-speaking children typically delete final consonants, and monolingual children speaking either language typically produce final consonants correctly between 3;0 and 4;0 (Dodd, Holm, Hua, & Crosbie, 2003; To et al., 2013). Carol also noted that English has many more consonants in final position (e.g., there are no word-final fricatives in Cantonese); thus, cross-linguistic transfer also may have impacted Tom's production of final consonants in English.

*Consonant clusters*. Because there are no consonant clusters in Cantonese, cross-linguistic transfer may have impacted Tom's production of consonant clusters in English.

*Backing.* Tom produced front plosives (stops) as back plosives (e.g., /t/ produced as [k]) in both languages. Backing of plosives is not typical for English-speaking children (Dodd et al., 2003); however, it does occur in typically developing monolingual Cantonese speakers up to age 3;6 (To et al., 2013). Therefore, cross-linguistic transfer from Cantonese may have impacted Tom's production of front plosives in English.

*Stopping.* Tom had difficulty producing fricatives in both English and Cantonese. Stopping is common in both languages but is not typical for monolingual Cantonese- or English-speaking children at age 4;11.

#### Diagnosis: Is There a Problem?

Tom has difficulty producing speech sounds in Cantonese and English. His parents rated that his speech is “sometimes” intelligible in Cantonese. His PCC scores were low even compared with younger Cantonese–English bilingual children with typical development. His patterns of error indicate some cross-linguistic transfer, but he also has errors that are not typical for Cantonese-speaking peers (e.g., stopping) or English-speaking peers (final consonant deletion and cluster reduction). Therefore, Tom probably has SSD and would benefit from intervention to enable him to be more intelligible as he begins school.

#### Goal Setting: What Support Does This Child (and Family) Need?

Carol decided that Tom would benefit from intervention in Cantonese and English. Tom's intervention goals were to reduce the occurrence of final consonant deletion, backing, and stopping. Carol worked with the parents and interpreters to generate a list of relevant words. For example, the following words were used to elicit fricatives: 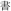 *book* /syu_1_/, 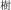 *tree* /syu_6_/, *sea* /si/ and, *sun* /sʌn/, and additional words were obtained from the book by McLeod and Baker (2017).

This case study demonstrates that SLPs have the ability and resources to undertake a speech assessment of multilingual children, including children who speak nondominant languages not shared by the SLP. SLP education programs should deliberately focus on recruitment of students who do not speak the dominant ambient language as their first language and provide coursework on multilingual and multidialectal assessment and intervention to support professional practice in an increasingly multilingual world.

## Footnotes

1 “Children with speech sound disorders can have any combination of difficulties with perception, articulation/motor production, and/or phonological representation of speech segments (consonants and vowels), phonotactics (syllable and word shapes), and prosody (lexical and grammatical tones, rhythm, stress, and intonation) that may impact speech intelligibility and acceptability” (International Expert Panel on Multilingual Children's Speech, 2012, p. 1).

2 Children with speech differences produce speech differently from those who speak the dominant language or dialect.

3 “People who are multilingual, including children acquiring more than one language, are able to comprehend and/or produce two or more languages in oral, manual, or written form with at least a basic level of functional proficiency or use, regardless of the age at which the languages were learned” (International Expert Panel on Multilingual Children's Speech, 2012, p. 1).

4 Prosody also differs between languages and dialects (Hirst & Di Cristo, 1998; Peppé et al., 2010).

5 Superscript “h” indicates aspirated sounds, and superscript “w” indicates lip-rounding co-articulation in the production of /k/ (International Phonetic Association, 2015).
